# Therapeutical and Pharmaceutical Notes

**Published:** 1901-11

**Authors:** W. J. Martin

**Affiliations:** Kankakee, Ill.


### THERAPEUTICAL AND PHARMACEUTICAL NOTES.

UNDER THE DIRECTION OF W. J. MARTIN, M.D.C.,
KANKAKEE, ILL.

QUININE EXTRACTION. As practised in Java, this simply consists of treating the powered bark with a 5 per cent. solution of caustic soda heated to 50° C., throwing the mechanically agitated mass into a reservoir containing Java petroleum, specific gravity 0.999, removing the petroleum solution of alkaloids by mechanical devices into a warm reservoir, into which is poured water acidulated with sulphuric acid. The aqueous layer is removed, evaporated, and the quinine sulphate separated from the concentrated solution by crystallization. This product contains only one-half per cent. of cinchonine. It is said that 50,000 kilos of this quinine are annually exported to the United States.—*Bulletin of Pharmacy.*

ADULTERATION OF CINNAMON BARK. Guava, or jungle bark, is reported (*Pharmaceutical Era*) to be used in Great Britain for the manufacture of imitation cinnamon. This bark is carefully peeled, prepared, and dried like cinnamon, and closely resembles it in appearance. The cinnamon odor is imparted to the bark by immersing it in water obtained from the distillation of cinnamon oil, and afterward, when dry, by touching the ends of each bundle of the bark with a cloth saturated with cheap cinnamon oil.

LIQUOR CARBON DETERGEN. This valuable preparation, so extensively used in human practice for the treatment of eczema and the various other forms of skin diseases, is prepared as follows: Mix 4 ounces of coal-tar with 8 ounces of tincture of soap-bark (quillaja saponaria), allowing the mixture to stand for a few days in a warm place, shaking occasionally and afterward filtering. It is in this strength used pure, being most generally mixed with water in the proportion of 1 drachm to 2 ounces to 1 drachm to 6 ounces of water. I believe that this preparation will be found of much benefit in the treatment of the many forms of eczematous types of skin diseases met with in veterinary practice.

PROF. ROBERT KOCH. This renowned German bacteriologist, who has lately created such a profound sensation throughout the entire world by his statement that bovine tuberculosis was not communicable to man, was born at Clausthal, Hanover, December 11, 1843. He studied medicine from 1862 to 1866, and graduated from the University of Göttingen. He began the practice of medicine at Langenhagen, and later removed to Racknitz, in Posen. From 1872 to 1880 he was district physician at the small village of Wollstien. His first important contribution to bacteriology was on anthrax, which was published in 1876 and attracted wide attention to the author. This was followed by a report on traumatic and infectious diseases, which was published in 1878. Professor Koch has been the recipient of the highest honors and decorations from nearly every civilized government in the world in recognition of his manifold benefits to humanity. After Louis Pasteur, Professor Koch has probably done more for the alleviation of sickness and pain, both among men and the animal kingdom, than any other person who has ever lived within the present historical period.

Tropacocaine Hydrochlorate. Myer (*Medical News*, April 13, 1901) states that tropacocaine was discovered by Giesel in 1891 in Javanese coca leaves, and that the alkaloid was more closely studied by Liebermann, who succeeded in producing it synthetically. Later, Willstatter discovered a way of preparing tropacocaine from tropine, a fractionation product of atropine and hyoscyamine. His method is the one now exclusively employed in the manufacture of the alkaloid. Since then the salt has been studied by many investigators, and the following points of importance in comparison to cocaine were found :

1. Tropacocaine is less than half as toxic as cocaine.
2. The depressing action, both on the cardiac motor ganglia and on the cardiac muscle, particularly the latter, is much greater with cocaine.
3. Recovery from its effects is much more rapid.
4. The solution is much more stable than that of cocaine hydrochlorate.

Absorption of Various Drugs by the Bladder. Barbiani (*Annales des Maladies des Organes Genito-urinaires*, February, 1901) has found by experimental research that dilute solutions of various drugs, with the exception of iodine, are never absorbed by the bladder at the first irrigation, but that if consecutive irrigations are made there is a fairly active absorption. This he attributes to certain functional disturbances of the epithelium incident to the mechanical action of the lavage upon the vesical walls, since such absorption may take place without further alteration of this epithelium. He believes that the complete retention of urine is in itself sufficient to produce such changes in the vesical mucosa as are calculated to encourage absorption.—*Therapeutic Gazette.*

New Turpentine Liniment. Oil of turpentine, 2 ounces; wool-fat or lanolin, 1 ounce; water, 7 ounces. Mix the wool-fat with an equal weight of oil of turpentine, and gradually add water, with constant stirring; incorporate in the mixture the remainder of the oil of turpentine, shaking or rubbing until a perfectly homogeneous liniment is formed.

Roup in Fowls. The following formula is recommended by the *Western Druggist* for roup or contagious catarrh of fowls: Capsicum, 2 drachms; potassium chlorate, 2 ounces; cubebs, 2 ounces; anise, 2 ounces; licorice-root, 2 ounces; Indian meal, 8 ounces. Mix one teaspoonful with the food for ten chickens. Roup pills: Copaiba, 1 ounce; cubebs, 1 ounce; licorice-root, 1 ounce; capsicum, 15 grains. Make into a suitable pill mass and divide into 5-grain pills, of which one is given twice a day. As a tonic during convalescence it is recommended to give Douglass' mixture. The formula for the latter is: Ferrous sulphate, 240 grains; dilute sulphuric acid, 30 minims; water, 16 ounces. A teaspoonful of this mixture is added to each quart of drinking-water for birds and poultry. Affected fowls are to be promptly isolated and disinfectants used. They are to be kept in a clean, warm, and dry place and fed on warm, soft feed; bowels are kept open with castor oil, and the inflamed and discharging membranes of the throat and nose and eyes are cleansed by sponging or injections with a diluted solution of chlorinated soda. If they die they should be burned.

A Comparison of Antiseptics. The following excerpts are taken from an excellent short article by Hooper on a comparison of antiseptics, in the *Canadian Practitioner and Review* for April, 1901, and are based on a report on the literature on the subject by the State Board of Health of Maine:

Reinicke, Ahlfeld, and Epstein, in an extended series of investigations, agree that the most important condition favoring the action of alcohol is the presence of moisture. It is, moreover, a valuable auxiliary, as Epstein's conclusions clearly show, "that absolute alcohol has no disinfecting power; that 50 per cent. disinfects better than higher or lower concentrations; that antiseptics which have more or less efficiency as aqueous solutions lose their disinfecting properties when dissolved in high-grade alcohol, but that, on the other hand, solutions of sublimate, carbolic acid, lysol, and thymol have a higher power of disinfection in 50 per cent. alcohol than solutions of the same concentrations in water have." In itself alcohol has no valuable antiseptic qualities, but is useful in that it enhances the antiseptic properties of other agents.

The convenient method of boiling is found to destroy in a few minutes most disease germs at a point considerably below boiling. Cholera spirillum was killed at a temperature of 125° F. in four minutes, typhoid bacillus at 139° F. in ten minutes, staphylococcus pyogenes aureus at 136.4° F. in ten minutes.

In a comparison of boiling water and steam the former has a distinct advantage in that it more readily absorbs moisture, and thus destroys the vitality of the bacteria. The same volume of steam encounters in penetrating bacteria a medium which is undoubtedly a coating of minute air-bubbles adherent to the germs. Plunged into water, air has a tendency to rise to the surface; this is due to the great difference in the specific gravity of air and water; the difference at 100° C. is about 1 to 1000; with steam it is only 3 to 5; the steam is deprived, therefore, of this valuable aid in freeing the bacteria from air-bubbles.

Carbolic acid is so universally relied on and adhered to by the medical profession that it is well to be aware of its limitations. Koch says for the destruction of anthrax spores a 3 per cent. solution must act seven days; the same percentage of these solutions separately did not destroy these spores in thirty days. Dr. Scheurlen, in a paper on the molecular conditions of aqueous solutions of disinfectants as regards their efficiency, states that a 1 per cent. solution of carbolic acid in water failed to destroy staphylococcus pyogenes aureus in five minutes, but a 1 per cent. solution of carbolic acid with 20 per cent. of common salt destroyed the same organisms in one minute. Upon Scheurlen's recommendation certain surgeons have used the ½ per cent. solution of orthocresol with 12 per cent. of common salt as a very satisfactory antiseptic. The rusting of instruments in it can be prevented by the addition of 1 to 1000 of hyposulphite of soda. It is well to emphasize Koch's statement and Lenti's confirmation that carbolic acid in olive oil or absolute alcohol has no effect whatever.

Lysol consists of neutral soap, water, and cresol. It is undoubtedly a better disinfectant than carbolic acid, and is also cheaper. Gruber found that a 2 per cent. solution of lysol destroyed the staphylococcus of suppuration as readily as a 3 per cent. solution of carbolic acid. In Martin's clinic in Berlin the statistical showing was more favorable after the use of lysol than after that of carbolic acid. Gerlack, in speaking of its advantages in surgical practice, says that lysol is more efficient than carbolic acid; that the disinfection of the hands is assured by using a 1 per cent. solution without the previous use of soap; that a 1 per cent. solution renders instruments sterile and does not attack the instrument, and that it is eight times less poisonous than carbolic acid, and much less than corrosive sublimate. The one disadvantage of lysol—namely, rendering the hands and instruments slippery—can be overcome by subsequent washing in sterilized water.

Solveol is a preparation of cresol held in aqueous solution by means of cresotinate of soda. It contains 27 per cent. of cresol, and is used principally as a surgical antiseptic. It forms clear and perfectly neutral solutions in water; solutions of the same strength are twenty times less poisonous and much less caustic than those of carbolic acid; its solutions do not roughen the hands, as corrosive sublimate does, nor render them slippery, as lysol does, nor obscure the field of operation, as the precipitate of creolin does; ts odor is less persistent than that of carbolic acid; diluted with calcareous waters, precipitates are not formed as with corrosive sublimate and lysol. It speaks most favorably for solveol that Hammer found 5 per cent. of solveol to act more energetically than a 2.5 per cent. solution of creolin, lysol, or carbolic acid.

The suitability or unsuitability of corrosive sublimate for certain disinfecting purposes is a question which has been widely discussed. The inability of a 1 to 1000 solution to destroy staphylococcus aureus in less than twenty-three hours is very unfavorable evidence against the sublimate. McClintock, after a series of experiments, is forced to the conclusion that the germicidal power of solutions of sublimate has been enormously overestimated. He closes with the following summary: The high rank heretofore given corrosive sublimate as a germicide is without warrant, and was based upon faulty experiments. Sublimate forms, with cellulose, milk, albuminous bodies, with some parts of bacteria—probably the envelope—a chemical compound that cannot be removed by any amount of washing with water. This sublimate, when acting on a germ, forms a capsule around it, which for a time protects the germ from the further action of the sublimate. Three objections exist to mercuric chloride being considered an ideal antiseptic in surgical work: Its great toxicity, which requires the greatest care in its use; its precipitation of albuminous material, and the tarnishing of instruments. A fourth may be added—that of roughening the hands.

To Dr. Charles McClintock we are indebted for the results of thousands of experiments performed to make an antiseptic soap in which mercurial salts remain in an active form and undecomposed. A solution of the double salt of mercury and potassium iodide was found to permit the presence; if a weak alkali is used the metals are attacked, and if too much the mercury is precipitated. The following conclusions express well the merits of antiseptic soap: 1. In proportion to the amount of antiseptics contained this soap is at least four times stronger than any known germicide. A 1 per cent. solution of the soap, 1 to 5000 of mercuric iodide, is at least equal to 1000 of mercuric chloride. 2. It does not coagulate albumins or attack nickeled or steel instruments. 3. It does not attack lead pipes or silver and aluminium instruments. A solution containing $\frac{1}{4}$ per cent. of soap, or 1 to 2000 of mercuric iodide, has to its credit the destruction of staphylococcus in one minute.—*Therapeutic Gazette.*

---

CHLORETONE AS A LOCAL ANÆSTHETIC. Rasely (*International Journal of Surgery*, April, 1901) has found chloretone equally as good as cocaine in operative cases, including the incision of abscesses, amputation of fingers, and even in the extraction of teeth without the least evidence of a depressing effect.

He has operated on a case of complete fistula in a man, aged seventy-four years, with fatty degeneration of the heart, using chloretone as the anæsthetic for several reasons. No pain was felt, and there was not the slightest manifestation of depression or exhilaration, although syringeful after syringeful of the saturated solution was used in the line of the proposed incision.

Rasely thinks chloretone an ideal local anæsthetic, and he believes it to be sure in effect and safe in results. He thinks it is well adapted to taking the place of cocaine in subarachnoid anæsthesia.—*Therapeutic Gazette.*

---

THE Veterinary Medical Association of New Jersey will hold its annual meeting at Trenton, January 9, 1902.

